# Impacts beyond primary outcomes: a mixed-methods study exploring multiple perspectives of a health system intervention in Eastern Uganda

**DOI:** 10.1186/1472-6963-14-S2-P29

**Published:** 2014-07-07

**Authors:** Deborah DiLiberto, Sarah Staedke, Catherine Maiteki-Sebuguzi, Susan Naiga, Clare Chandler

**Affiliations:** 1 Department of Medical Statistics, London School of Hygiene & Tropical Medicine, London, UK; 2 Infectious Diseases Research Collaboration, Uganda, Uganda; 3 Department of Global Health and Development, London School of Hygiene & Tropical Medicine, London, UK; 4 Department of Clinical Research, London School of Hygiene & Tropical Medicine, London, UK

## Background

Interventions aiming to improve health systems should engage people on the front lines of health care delivery. Evaluations of these interventions should focus on the multiple change processes and outcomes resulting from their implementation into dynamic social systems. The PRIME intervention was designed to build health workers’ (HWs) skills by supporting and motivating them emotionally in their challenging work environments with the goal of improving treatment and attracting patients to health centres (HC) in Eastern Uganda. We conducted a cluster-randomised trial (CRT) to evaluate the impact of PRIME on health outcomes in the community and a parallel mixed-methods study to examine the effect of PRIME from the perspective of HWs and patients enrolled in the trial.

## Methods

Twenty HCs were enrolled in the CRT; 10 were randomized to the intervention with the primary endpoint of health outcomes measured in community-level clusters over two years. Mixed-methods included 306 HW communication assessments investigating the change in HW interpersonal skills with patients, 10 in-depth interviews exploring HWs interpretations and enactment of the intervention, 13 focus group discussions with community members discussing perceptions of change relating to PRIME, and 1200 patient exit interviews at HCs over three time points assessing patients’ satisfaction with their treatment seeking experience.

## Results

Post PRIME implementation, mixed-methods evaluations revealed that interpersonal communication was rated 10% higher (p<0.008) by patients consulting with HWs in intervention HCs. HWs revealed that improvement of technical skills and use of new technologies had a positive effect by increasing feelings of professionalism coupled with patients’ positive feedback; however, HWs also felt unsupported in other aspects including increased workload, and lack of recognition, payment and supervision leading to demotivation. Patients reported increased satisfaction with certain aspects of the treatment seeking experience, but also highlighted other areas of HCs needing improvement.

## Conclusion

CRTs of health system interventions focus on assessing the intended impact the intervention using a singular primary endpoint evaluation. Our results reveal that despite a lack of significant effect in the CRT primary health outcomes, the mixed-methods study demonstrated impacts including benefits, consequences, motivations, and interpretations from the perspective of the people who are central to the health system dynamic PRIME was intending to change. We will discuss what can and cannot be achieved and brought to light through a CRT model of evaluation of people-centred health system interventions and what this means for informing the design and implementation of future health system interventions.

